# Factors associated with relapse into drug use among male and female attendees of a three-month drug detoxification–rehabilitation programme in Dhaka, Bangladesh: a prospective cohort study

**DOI:** 10.1186/1477-7517-10-14

**Published:** 2013-09-01

**Authors:** Yuki Maehira, Ezazul Islam Chowdhury, Masud Reza, Ronald Drahozal, Tarun Kanti Gayen, Iqbal Masud, Sonia Afrin, Noboru Takamura, Tasnim Azim

**Affiliations:** 1 Department of Global Health, Medicine and Welfare Atomic Bomb Disease Institute, Nagasaki University, 1-12-4 Sakamoto, Nagasaki 852-8523, Japan; 2 International Centre for Diarrhoeal Disease Research, Bangladesh (icddr,b), 68 Shaheed Tajuddin Ahmed Sarani, Mohakhali, Dhaka 1212, Bangladesh; 3 Ashokti Punorbashon Nibash (APON), 9/7 Iqbal Road Mohammadpur, Dhaka 1207, Bangladesh; 4 The Society for Community Health, Rehabilitation, Education and Awareness (CREA), 1/14 Iqbal Road, Mohammadpur, Dhaka 1207, Bangladesh; 5 Dhaka Ahsania Mission (DAM), Dhanmondi R/A, Dhaka 1209, Bangladesh; 6 CARE Bangladesh, 20-21 Kawran Bazar, Dhaka 1215, Bangladesh

**Keywords:** Relapse, Gender, Drug detoxification-rehabilitation, People who use drugs, Bangladesh

## Abstract

**Background:**

To determine relapse rates and associated factors among people who use drugs (PWUDs) attending abstinence-oriented drug treatment clinics in Dhaka, Bangladesh.

**Methods:**

A cohort of male and female PWUDs admitted to the 3-month drug detoxification-rehabilitation treatment programmes of three non-governmental organisation-run drug treatment clinics in Dhaka, Bangladesh were interviewed on admission and over the following 5 months, which included the first 2 months after discharge. The study subjects comprised 150 male and 110 female PWUDs who had been taking opiates/opioids, cannabis or other drugs (including sedatives) before admission, had provided informed consent and were aged ≥16 years. Interviews were conducted using semi-structured questionnaires at four time points; on admission, at discharge and at 1 and 2 months after discharge. Relapse rates were assessed by the Kaplan–Meier method. Factors associated with relapse on enrolment and after discharge were determined using the Cox proportional hazards regression model.

**Results:**

A greater proportion of female than male subjects relapsed over the study period (71.9% versus 54.5%, *p* < 0.01). For men, baseline factors associated with relapse were living with other PWUDs (relative hazard ratio [RHR] = 2.27), living alone (RHR = 2.35) and not having sex with non-commercial partners (RHR = 2.27); whereas for women these were previous history of drug treatment (RHR = 1.94), unstable housing (RHR = 2.44), higher earnings (RHR = 1.89), preferring to smoke heroin (RHR = 3.62) and injecting buprenorphine/pethidine (RHR = 3.00). After discharge, relapse for men was associated with unstable housing (RHR = 2.78), living alone (RHR = 3.69), higher earnings (RHR = 2.48) and buying sex from sex workers (RHR = 2.29). Women’ relapses were associated with not having children to support (RHR = 3.24) and selling sex (RHR = 2.56).

**Conclusions:**

The relapse rate was higher for female PWUDs. For both male and female subjects the findings highlight the importance of stable living conditions. Additionally, female PWUDs need gender-sensitive services and active efforts to refer them for opioid substitution therapy, which should not be restricted only to people who inject drugs.

## Introduction

In Bangladesh, there is a long history of illicit drug use, particularly of cannabis and opiates [1]. The drugs commonly used have changed over time: heroin became very popular in the early 1990s but, because it was in short supply in the mid-1990s, other drugs were introduced [1-3]. Pharmaceuticals, in particular buprenorphine, are commonly injected, often with anti-histamines and diazepam. Injection of pethidine (meperidine) does occur, but is less common. Heroin is rarely injected [3]; however, heroin smokers intermittently inject or sometimes switch to injecting other drugs for various reasons [4,5]. At present multiple drugs are available and most people who use drugs (PWUDs) are poly drug users [4]. As of 2009, it is estimated that there are 21,800–23,800 people who inject drugs (PWIDs) in Bangladesh [6]. Available information suggests that there are fewer female than male PWUDs [4,7], and that they are mostly hidden and very mobile. Most female PWUDs who have been accessed are primarily non-injectors who prefer to smoke and inject infrequently [4].

Harm reduction services for male PWIDs were initiated in Dhaka in the late 1990s. These services include a needle and syringe programme (NSP), treatment for sexually transmitted infections (STIs), abscess management, provision of rest and recreational facilities, HIV/AIDS prevention education and distribution of male condoms. The harm reduction services are operated primarily through outreach workers and drop-in centres located within the community. The NSP in Bangladesh has been recognised as best practice by the Joint United Nations Programme on HIV/AIDS [8]. These services, which have now been expanded to cover different parts of Bangladesh, reached approximately 60% of PWIDs in 2008 [9], when Bangladesh had the widest NSP coverage in south Asia [10]. As of 2012, approximately 14,000 PWIDs are covered under the NSP of Bangladesh, including 400–500 women (unpublished data).

Access to affordable and good-quality drug treatment is a common demand emanating from PWIDs [11]. However, standardised drug detoxification and rehabilitation services are not readily available in Bangladesh. As of 2008, eight government clinics were being operated by the Department of Narcotics Control and Directorate of Prisons of the Government of Bangladesh and 162 treatment centres by private and non-governmental organisations (NGOs) [9] Services provided through the non-government sector are too expensive for the vast majority of PWUDs in Bangladesh [4,10]. It is believed that the NGO-run treatment programmes provide a wider range of services, such as rehabilitation with health education, behavioural change communication and life skills development, in addition to detoxification. However, these clinics do not follow any standard format and it is difficult to gauge the success of their programmes. In response to the need for good-quality and affordable treatment for PWUDs, in August 2005, Family Health International (FHI) established 10 drug treatment clinics in different areas of Bangladesh, three of which are in Dhaka, the capital city. These clinics have a standard 3-month programme of detoxification and rehabilitation.

Although there is evidence that opioid substitution therapy (OST) is both an effective harm reduction strategy and a form of drug treatment and does reduce relapse rates [12], Bangladesh had no OST programmes until 2010 [13], when one was approved as a pilot project. Since this programme’s initiation in 2010, more than 180 adult PWIDs have been enrolled, including eight women who had failed abstinence-based treatment at least twice (unpublished data). The pilot study had positive outcomes in terms of reducing risk behaviours, improving quality of life and improving physical and mental status. Based on these results, the pilot study has been scaled up to include 600 PWIDs over the next 3 years. However, it will still cover only a small proportion of PWIDs and only those who inject; none of those who use opioids but do not inject.

Relapse following drug treatment is common. It has been reported globally even in countries with high rates of completion of inpatient treatment: 33% in Nepal [14], 55.8% in China [15] and 60% in Switzerland [16] relapsed into drug use between 1 month and 1 year after discharge from treatment programmes. Multiple factors, such as post-treatment incarceration, mental or other comorbid disorders, craving for drugs and withdrawal symptoms, are reportedly associated with relapse [17,18]. In Bangladesh there are no reliable data on relapse following drug treatment. Anecdotal evidence suggests that 60 – 90% of patients eventually relapse.

To gain a better understanding of relapse rates and factors associated with relapse in Bangladesh, we conducted a prospective study on male and female PWUDs in settings where standardised, abstinence-oriented treatment and rehabilitation services were being provided.

## Methods

### Sampling site and study population

Male and female PWUDs were enrolled on admission to the three FHI-supported NGO drug treatment clinics in Dhaka. These NGOs are Ashokti Punorbashon Nibash, the Society for Community Health, Rehabilitation, Education and Awareness, and Dhaka Ahsania Mission. These FHI-supported residential and abstinence-oriented treatment programmes are provided free of cost. The programme consists of an initial 14-day detoxification followed by a 3-month rehabilitation programme at the same residential setting. During detoxification, manifestations of withdrawal are controlled by clonidine (alpha-2 adrenergic agonist), taking appropriate precautions and monitoring of concomitant medical conditions. During the 2-week detoxification period, PWUDs are not permitted to leave the residential facilities but are allowed visitors once a week and can use the telephone in the case of an emergency. After completing the 2 weeks of detoxification, PWUDs are permitted to return to their homes if they so desire. The decision to participate in the residential rehabilitation programme is voluntary and they can return to do so whenever they are ready. During the 3 months of residential rehabilitation, PWUDs are allowed to visit their homes in their villages or towns only as a rehabilitation programme activity to develop their coping skills.

While at the treatment centre, PWUDs receive regular health check-ups, clinical care and counselling (with or without family members). Towards the end of the programme, they are provided life skills and vocational training. Psychotherapeutic support, including management of craving and peer pressure, anger control, meditation and prayer, is provided by counsellors. PWUDs are also given basic education on sexual health including HIV/AIDS and STI prevention and drug-related harm. Physicians are available 6 days a week to respond to manifestations of drug withdrawal or uncomplicated psychiatric problems. On completion of the 3-month programme, the PWUDs are discharged and can return to their communities. Table 1 shows the daily schedule of the rehabilitation programme at the three NGO-run clinics.

**Table 1:** Daily schedule of NGO-run drug detoxification-rehabilitation programme in Dhaka, Bangladesh


**Detoxification period: (14 days)**	• Before and immediately after admission, the clinic center rules and regulations, service details are informed to the client.	
	• The program schedule is flexible for the client as they struggle with withdrawal symptoms and are not fit enough physically and mentally.	
	• Close supervision by medical professionals is ensured for the client during this period based on the treatment protocol that covers pharmacological withdrawal control, monitoring and precautions, other symptomatic medications applicable at clinics.	
	• Motivational sessions are offered to the client after 7 days to learn benefits of drug free lives, risk of drug use, HIV/STI, Tuberculosis.	
	• After two weeks of detoxification period, the clients are fully involved as shown in the schedule below of the rehabilitation program:	
**Time**	**Session/activities**	**Psychosocial and clinical course content**
6:15–6:45	Wake up and morning prayer	Prayer (within the group)
6:45–7:00	Exercise	Freehand exercise and YOGA
7:00–7:45	Housework	Occupational therapy
7:45–8:30	Wash/refreshing & group work	
8:30–9:15	Breakfast & medicine	
9:15–9:45	Quiet time/relaxation & pre meeting	Relaxation and meditation
9:45–10:30	Morning meeting/self evaluation	Therapeutic community’s morning meeting for community life/individual weekly evaluation
10:30–10:45	Free or personal time/(bathroom, wash)	Personal hygiene and care
10:45–11:30	Morning session	Addiction, anger management, post acute withdrawal management, building self-esteem, values, HIV & STI, communication
11:30–12:00	Tea break	
12:00-12-30	Occupational therapy/crafts work/routine health check-up	Development of habit for daily routine/health check-up
12:30–1:15	Wash/bath time	Personal hygiene and care
1:15–1:45	Prayer time & Talim (preaching)	Religious practice
1:45–2:45	Lunch & medicine, rest	
2:45–3:30	Evening session	Planning, relapse prevention, group therapy, psycho therapy, resentment, basics of behavior change
3:30–3:45	Routine work/house work	Occupational therapy
3:45–4:00	Prayer time	Religious practice
4:00–4:15	Tea break	
4:15–5:15	Sports & recreation	Outdoor games. summer- football, volleyball. winter- cricket, badminton
5:15–5:30	Prayer time	
5:360–6:15	Quite time	
6:15–7:00	Narcotics anonymous meeting (sharing meeting)	Verbalize and ventilate emotion, feeling, experience , etc.
7:00–8:00	Group meeting	Community meeting
8:00–8:30	Prayer time	
8:30–9:00	Dinner & medicine	
9:00–9:50	Free time/recreation/watching TV	
9:50–10:00	Bed preparation & self-cleaning	
10:00–10:45	Night sharing	Sharing and moral inventory
10:45	Bed time and lights off	

In this study, male and female PWUDs who were ≥16 years of age, fulfilled the clinical entry criteria set by the FHI and the clinics and provided informed consent were enrolled at the time of their admission to these clinics. The clinical criteria for admission were readiness for treatment and absence of severe comorbidities, including diabetes mellitus, liver disorders, epilepsy, psychiatric disorders and cardiac or respiratory problems. The reason for exclusion of PWUDs with psychiatric disorders was that no psychiatrists were available at the clinics. However, if PWUDs were diagnosed with a psychiatric disorder after admission, they were referred to psychiatrists at other institutions. In addition, pregnant women were not admitted because the clinics did not have the facilities and staff for adequate handling of obstetric emergencies.

In this study, the PWUDs included both those who injected (i.e. PWIDs) and those who smoked heroin. PWIDs were defined as those for whom the main route of drug administration was injection and who had injected at least once in the past 6 months. Heroin smokers were defined as those whose drug of choice was heroin, whose main route of drug administration was inhalation (smo king, chasing or snorting), and who had not injected drugs more than twice in the last 6 months.

### Study design

All enrolled PWUDs had one-to-one interviews using semi-structured questionnaires at four time points over a period of 5 months to determine risk behaviours. The interviews were conducted by trained male and female interviewers on admission to and discharge from the clinics and twice in the community, 1 and 2 months after discharge. Those who dropped out of the treatment programme were interviewed at the point of dropout (before leaving the clinic) where possible, or in the community after dropout as soon as they had been located.

Prior to enrolment written informed consent was obtained from all PWUDs. The study was approved by the Research and Ethical Review Committees of the International Centre for Diarrhoeal Diseases Research, Bangladesh.

### Determination of characteristics of enrolled PWUDs, risk behaviour and relapse

The semi-structured questionnaire was designed to ascertain relevant socio-demographic characteristics, history of drug use, HIV risk behaviours related to drugs and unsafe sex and reasons for relapse following treatment.

Relevant socio-demographic characteristics of PWUDs included where they lived, who they lived with and their income. Where they lived was classified according to the UN-Habitat definitions [19] as shown below:

•House: a durable structure, independently built and with access to adequate sanitation such as private toilets and safe water.

•Working place: includes shops, offices, clubs or wherever the person works that provides adequate shelter with access to toilets.

•Street: an open space with minimal shelter such as under awnings, in abandoned buildings, verandas and bus/train terminals.

•Slum: a household shared by a group of individuals in an urban area characterised by substandard, non-durable housing and shared basic sanitation facilities.

These categories were regrouped into unstable housing (unsheltered locations categorised above under “street”) and stable housing (all other locations, namely house, working place and slum).

For drug use characteristics and injection-related risk behaviours, questions were asked about preferred choice of drugs, route(s) of administration and injection practices (whether interviewees had borrowed or lent used needles/syringes; number of sharing partners in the last sharing episode and frequency of sharing). For sexual risk behaviours, questions were asked about history of sex with non-commercial and commercial sex partners, condom use during last sex and frequency of condom use.

The point of relapse was defined as the first time that drugs were used after discharge or dropout from the treatment programmes. This was determined by the response to the question “When did you first take drugs after discharge (or dropout)?”, which was asked when PWUDs were interviewed in the community 1 and 2 months after discharge. The single open-ended question “What was your reason for starting drug use again after discharge?” was asked to determine the reasons for relapse and the responses allocated to one of the following five categories:

•Peer influence.

•Family-related problems such as loneliness, poverty, family disputes and abuse by family members.

•Personal problems such as lack of job or money, concern about own pregnancy, legal case, problems with lover/girlfriend or boyfriend, dropping out of school or work and abuse by members of their local communities.

•Drug craving, including withdrawal symptoms.

•To enhance sexual performance.

Additionally, the following questions were asked to determine whether PWUDs perceived any changes in their drug use pattern following drug treatment and what those changes were: “Do you think your current drug use habit is different from that before your admission?” and “What is different from your previous drug use pattern?”

### Statistical analyses

Enrolled PWUDs were divided into two groups, male and female PWUDs. Data were entered using Epi-Info (Version 3, Centres for Diseases Control and Prevention, Georgia, USA) and analysed using the Statistical Package for Social Sciences (SPSS) Version 15 (IBM Corporation, New York, USA). To determine whether there were differences between male and female PWUDs in baseline characteristics assessed on enrolment, these characteristics were compared between male and female PWUDs using the χ^2^ test for categorical data and the Mann–Whitney U test for nonparametric data. The Kaplan–Meier method was used to construct curves of time to relapse and determine median times to relapse for male and female PWUDs who completed the detoxification-rehabilitation programme and were followed up after discharge for at least 1 month. The log-rank test was used to compare times to relapse between male and female PWUDs [20].

Relapse may be associated with characteristics that PWUDs had before admission to the treatment programme (determined during admission interviews) or with factors that influence them after they return to their communities (determined through interviews conducted in the community after discharge). Therefore, data from two time points, namely on admission and after discharge, were subjected to bivariate analysis using the univariate Cox proportional hazards model to determine factors associated with relapse separately for male and female PWUDs [21]. Variables found to be significant at the 5% level by bivariate analysis were subjected to a multivariate backward stepwise Cox proportional hazards regression to determine the net effect of the factors associated with relapse. The effects of risk factors on relapse are expressed as relative hazard ratios (RHR) with their corresponding 95% confidence intervals (CI) and *p*-values. Covariates with *p*-values of ≤ 0.05 were considered to be significant.

## Results

### Response rate

**Figure 1:**
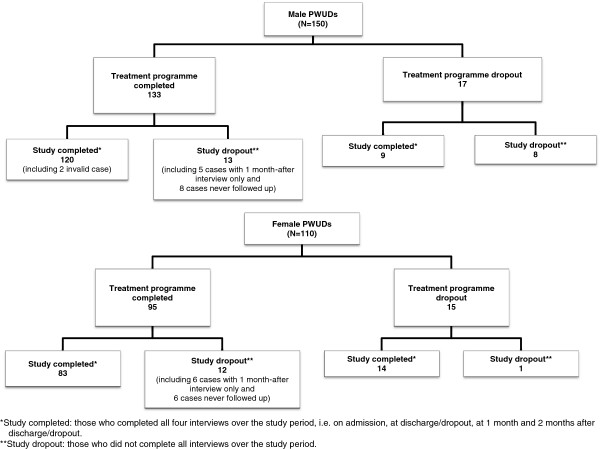
**Flow diagram showing number of subjects at various stages of the study.** Two flow diagrams to describe numbers of male (N=150) and female (N=110) study participants from respetive enrollment at admission until the study completion at the two months follow-up interview after discharge.

Figure 1 is a flow diagram showing numbers of subjects at various stages of the study. In all, 150 male and 110 female PWUDs were enrolled from January 2008 to February 2009 and 133 men and 95 women completed the 3-month treatment programme. Among those who completed treatment, 120 men and 83 women also completed the study. Of those who did not complete the study; five male and six female subjects were followed up for only 1 month and eight men and six women could not even be reached at 1 month after discharge. The reasons for loss to follow-up were not having supplied a valid address (10 men and 10 women), death (one woman) and imprisonment after discharge (one woman and three men).

The reasons for dropping out of the treatment programme were varied and included family reasons (one man and three women), health issues (two men and two women developed chicken pox), pregnancy identified after admission (two women) and inability to adjust to the treatment programme (three men and three women).

Thus, valid data for analyses related to relapse were available from 123 male and 89 female subjects.

### Baseline characteristics of drug users (on admission to clinics)

Table 2 shows the baseline characteristics of male and female PWUDs during the 6 months prior to admission.

**Table 2:** Comparison of baseline characteristics of PWUDs (on admission to clinics)

**Characteristics**	**Male PWUDs**	**Female PWUDs**	***p***
	**(N = 150, unless otherwise stated)**	**(N = 110, unless otherwise stated)**	
**Socio-demographic factors**			
Had previous experience of drug treatment programmes	54.0 (81)	67.3 (74)	0.03
Age in years			
Mean (SD)*	32.6 (±7.0)	26.8 (±6.4)	
Median (IQR)^▲.^	32.0 (27.0, 36.3)	25.0 (22.0, 30.0)	<0.01
Level of education, % (n)			
Less than 1 year	10.7 (16)	19.1 (21)	0.05
1–5 years	64.0 (96)	61.8 (68)	NS
>5 years	25.3 (38)	19.1 (21)	NS
Marital status, % (n)			
Married / living with sex partner	31.3 (47)	43.8 (49)	0.03
Unmarried	36.0 (54)	10.9 (12)	<0.01
Divorced/separated/widower/widow	32.7 (59)	44.5 (49)	0.05
Income level (monthly average in BDT^△^)			
Mean (SD)*	6144 (±3997)	11397 (±8750)	
Median (IQR) ^▲.^	5000 (3500, 7000)	7900 (5000, 15250)	<0.01
% more than Mean	33.7	38.2	
Locations where they live, % (n)			
House	48.7 (73)	30.9 (34)	<0.01
Working place (shop, office, club)	4.0 (6)	4.5 (5)	NS
Street^§^	42.0 (63)	20.9 (23)	<0.01
Slum	5.3 (8)	43.6 (48)	<0.01
Person(s) with whom they usually live, % (n)			
Family member, relative	40.7 (61)	34.5 (38)	NS
Spouse, sex partner	14.0 (21)	16.3 (18)	NS
Non-PWUD friend, colleague	2.6 (4)	6.3 (7)	NS
PWUD friend, drug dealer	26.7 (40)	29.1 (32)	NS
Alone	16.0 (24)	13.6 (15)	NS
Have children to support, % (n)	10.7 (16)	13.6 (15)	NS
**History of drug misuse (in the last six months before admission)**			
Drug primarily preferred for consumption, % (n)			
Cannabis, sleeping pills, codeine containing cough syrup, alcohol	1.3 (2)	13.6 (15)	<0.01
Heroin	16.7 (25)	72.8 (80)	<0.01
Buprenorphine, pethidine	82.0 (123)	13.6 (15)	<0.01
Main route of drug administration, % (n)			
Orally	0.0 (0)	3.6 (4)	0.03
Inhaling (smoking, snorting, chasing)	13.3 (20)	82.7 (91)	<0.01
Injection	86.7 (130)	13.6 (15)	<0.01
Duration (years) of injecting drugs (among those who injected in the last six months)	**N = 144**	**N = 26**	
Mean (SD)*	5.9 (±4.4)	4.1 (±4.7)	
Median (IQR) ^▲.^	5.0 (3.0, 8.0)	2.5 (1.0, 6.25)	0.01
% more than mean	34.0 (60)	35.6 (9)	
Shared used needle/syringe during last injection (among those who injected in the last six months), % (n)	**N = 144**	**N = 26**	
	54.2 (78)	34.6 (9)	NS
Shared injection paraphernalia during last injection (among those who injected in the last six months), % (n)	**N = 144**	**N = 26**	
	91.7 (132)	42.3 (11)	<0.01
**Sexual behaviour (in the last six months before admission)**			
Had any type of sex, % (n)	58.0 (87)	70.0 (77)	0.05
Bought sex from sex worker, % (n)	30.7 (46)	5.5 (6)	<0.01
Sold sex in exchange of money or drugs, % (n)	0	49.1 (54)	<0.01
Frequency of using condoms during sex with commercial sex partner (among those who bought or sold sex), % (n)	**N = 46**	**N = 54**	
Always	63.0 (29)	42.6 (23)	0.04
Sometimes	13.1 (6)	40.7 (22)	<0.01
Never	23.9 (11)	9 (16.7)	NS
Had sex with non-commercial partner, % (n)	35.3 (53)	45.4 (50)	NS
Frequency of using condom during sex with non-commercial partner (among those who had sex with non-commercial partner), % (n)	**N = 53**	**N = 50**	
Always	20.8 (11)	16.0 (8)	NS
Sometimes	22.6 (12)	18.0 (9)	NS
Never	56.6 (30)	66.0 (33)	NS
Had group sex, % (n)	2.7 (4)	19.1 (21)	<0.01
Had sex just after taking drugs, % (n)	45.3 (68)	50.9 (56)	NS
**Other risk behaviours (before admission)**			
Ever been imprisoned, % (n)	60.7 (91)	41.8 (46)	<0.01
Ever sold blood, % (n)	27.3 (41)	13.6 (15)	0.01

*SD Standard Deviation, ^▲.^*IQR* inter-quartile range, *NS* not significant (p>0.05).

^△^1 USD is equivalent to 75.88 BDT as of October 2011.

^§^Street includes abandoned place, park, roof-top, veranda, terminal, and garage.

Most PWUDs enrolled in this study provided a previous history of attending drug treatment programmes but this was more common in female subjects (men: 54%, women: 67.3%, *p* < 0.05). The women were younger than the men (*p* < 0.01), more were currently married or living with a partner (*p* < 0.05) and their median monthly earning was 1.6 times greater than that of the men (*p* < 0.01). The locations where they usually lived differed significantly between men and women; more women lived in slums (*p* < 0.01) whereas the men more often lived in their own houses (*p* < 0.01) or on the streets (*p* < 0.01). More than 85% of the enrolled drug users had no childcare needs.

The majority of the women preferred to inhale heroin, whereas the men preferred to inject pharmaceuticals; the same trend was seen in preferred route of drug administration. Among PWIDs (144 men and 26 women), there were no differences in the proportions of men and women who said they had shared needles/syringes during their most recent injection. However, men were more likely to share other injection paraphernalia (91.7% for men versus 42.3% for women, *p* < 0.01).

Regarding sexual behaviour, more female PWUDs reported engaging in commercial sex: 30.7% of men bought sex from sex workers, whereas almost half the women sold sex in exchange for drugs or money (*p* < 0.01). Six women, including three who sold sex, also reported buying sex. In addition, group sex was more commonly reported by women (*p* < 0.01). More men than women reported consistent use of condoms during commercial sex (*p* = 0.04). More than 45% of both male and female subjects reported having sex under the influence of drugs; this did not differ between the two groups.

Men more commonly reported having ever been imprisoned or selling blood than did women (*p* < 0.01 for both factors).

### Relapse among drug users who completed the 3-month treatment programme

Relapse rates and median time to relapse were calculated for the 123 male and 89 female PWUDs who completed treatment and for whom date of relapse was available. Of these subjects, 118 men and 83 women had completed all interviews throughout the study, whereas five men and six women could not be located for the second post-discharge interview (Figure 1).

**Figure 2:**
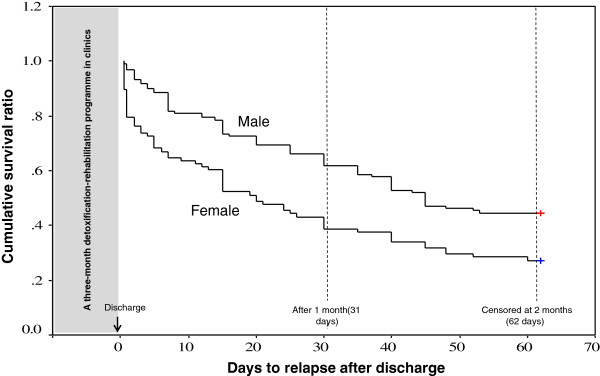
**Relapse among PWUD who completed the three-month treatment programme.** Survival curve to compare cumulative survival ratio of male and female drug users who relapsed within two months (62 days) after discharge from a three-month detoxification-rehabilitation programme at three NGO-run clinics.

Higher relapse rates were recorded for women than for men over the 2-month post-discharge observation period (71.9% versus 54.5%, *p* < 0.01) (Figure 2). Median times to relapse were 45 days for men and 20 days for women. Most of the relapses occurred during the first 30 days after discharge in both sexes.

### Factors associated with relapse in male and female PWUDs

The baseline covariates associated with relapse differed between male and female PWUDs (Table 3). Men were more likely to relapse if they were living with other PWUD friends or drug dealers (RHR = 2.27, *p* < 0.01), living alone (RHR = 2.35, *p* = 0.02) or not having sex with non-commercial partners (RHR = 2.27, *p* = 0.01). Women were more likely to relapse if they had previously attended other drug treatment programmes (RHR = 1.94, *p* = 0.03), lived in unstable housing (RHR = 2.44, *p* = 0.01), had incomes higher than the monthly average (RHR = 1.89, *p* = 0.02) or preferred to smoke heroin (RHR = 3.62, *p* = 0.01) or inject buprenorphine or pethidine (RHR = 3.00, *p* = 0.05) rather than take cannabis, sleeping pills, codeine containing cough syrup or alcohol.

**Table 3:** Baseline covariates* (before admission) associated with relapse of male and female PWUDs

**Covariate**	**Male PWUDs (N = 123)**			**Female PWUDs (N = 89)**		
**Category**	**B**	***p***	**Adjusted RHR (95% CI)**	**B**	***p***	**Adjusted RHR (95% CI)**
**Socio-demographic factors**						
Had previously undergone drug treatment				0.66	0.03	1.94 (1.08−3.49)
Location where they live						
Stable housing^§^						1.00 (Ref.)
Unstable housing†				0.89	0.01	2.44 (1.24−4.81)
Person(s) with whom they usually live						
Family member, relative			1.00 (Ref.)			
Spouse, sex partner	−0.10	0.85	0.91 (0.32−2.55)			
Non-PWUD friend, colleague	1.06	0.09	2.89 (0.84−9.90)			
PWUD friend, drug dealer	0.82	<0.01	2.27 (1.25−4.12)			
Alone	0.85	0.02	2.35 (1.18−4.68)			
Income level						
More than the monthly average ‡				0.64	0.02	1.89 (1.13−3.17)
**History of drug misuse (in the last six months before admission)**						
Type of drug used as the first choice						
Cannabis, sleeping pills, codeine containing cough syrup, alcohol						1.00 (Ref.)
Heroin				1.29	0.01	3.62 (1.39−9.46)
Buprenorphine, Pethidine				1.10	0.05	3.00 (1.02−8.81)
**Sexual behaviour (in the last six months before admission)**						
Not had sex with non-commercial partner	0.82	0.01	2.27 (1.19−4.33)			

*Only the covariates with significance of at least a 5% level for association with relapse were analysed by backward stepwise Cox multivariate analyses.

§Stable housing = house, working place, shop, office, club.

†Unstable housing = street, abandoned place, park, roof-top, veranda, bus/train terminal.

‡Monthly average incomes are BDT 6144 for male, and BDT 11397 for female (1USD is equivalent to 76.20 BDT as of 31 October 2011).

B: Net effect of independent variable. *RHR* relative hazards risk, *CI* confidence interval.

At post-discharge follow-up (Table 4), relapse of men was associated with four factors: unstable housing (RHR = 2.78, *p* = 0.04), living alone (RHR = 3.69, *p* = 0.02), having higher incomes than the mean monthly average (RHR = 2.48, *p* = 0.01) and buying sex from sex workers (RHR = 2.29, *p* = 0.01). Women’s relapses were associated with not having children to support (RHR = 3.24, *p* < 0.01) and selling sex in exchange for money or drugs (RHR = 2.56, *p* < 0.01).

**Table 4:** Follow-up covariates* (1 month after discharge) associated with relapse of male and female PWUDs

**Covariate**	**Male PWUDs (N = 123)**			**Female PWUDs (N = 89)**		
**Category**	**B**	***p***	**Adjusted RHR (95% CI)**	**B**	***p***	**Adjusted RHR (95% CI)**
**Socio-demographic factors**						
Location where they live						
Stable housing^§^			1.0 (Ref.)			1.0 (Ref.)
Unstable housing†	1.02	0.04	2.78 (1.05−7.35)	−0.68	0.06	0.51 (0.25−1.02)
Person(s) with whom they usually live						
Family member, relative			1.0 (Ref.)			
Spouse, sex partner	−0.25	0.56	0.78 (0.34−1.79)			
Non-PWUD friend, colleague	−0.83	0.05	0.44 (0.19−0.99)			
PWUD friend, drug dealer	0.06	0.93	1.06 (0.25−3.95)			
Alone	1.31	0.02	3.69 (1.29−10.53			
Income level						
More than monthly average ‡	0.91	0.01	2.48 (1.22−5.02)			
Not having children to support				1.18	<0.01	3.24 (1.38−7.64)
**Sexual behaviour (in the past 1 month)**						
Bought sex from sex workers	0.83	0.01	2.29 (1.25−4.20)			
Sold sex in exchange of money or drugs				0.94	<0.01	2.56 (1.52−4.29)

*Only the covariates with significance of at least a 5% level for association with relapse by backward stepwise Cox multivariate analyses.

§Stable housing = house, working place, shop, office, club.

†Unstable housing = street, abandoned place, park, roof-top, veranda, bus/train terminal.

‡Monthly average incomes are BDT 6144 for male, and BDT 11397 for female (1USD is equivalent to 76.20 BDT as of 31 October 2011).

B: Net effect of independent variables. *RHR* relative hazards risk, *CI* confidence interval.

### Perceived reasons for relapse and changes in drug use patterns after drug treatment

The two most common reasons for relapse were peer influence and family-related stress (Table 5). There were no differences between men and women in this regard. It is notable that, although women had a higher relapse rate (Figure 2), more women than men reported changes in their drug taking behaviour in that they had decreased the frequency or amount of drug consumed (*p* < 0.05).

**Table 5:** Perceived reasons for relapse and changes in drug use patterns 2 months after discharge from a 3-month drug detoxification-rehabilitation programme

**Characteristics**	**Male PWUDs N = 67, unless otherwise stated**	**Female PWUDs N = 64, unless otherwise stated**	***P***
**Reason to relapse, % (n)**	**(N = 67)**	**(N = 64)**	NS^*^
Peer influence (previous PWUD friends, sexual partner’s drug use)	32.8 (22)	45.3 (29)	NS
Family-related problem (no peace, poverty in family)	35.8 (24)	35.9 (23)	NS
Personal problems	19.4 (13)	14.1 (9)	NS
Drug craving, withdrawal symptoms (including restlessness, sleeping disturbance)	9.0 (6)	4.7 (3)	NS
To enhance sexual power	3.0 (2)	-	
**Changes perceived in current drug use pattern, % (n)**	**(N = 67)**	**(N = 64)**	0.02
	59.7 (40)	79.7 (51)	
**Major difference from previous drug use pattern (among those who perceived changes), %(n)**	**(N = 40)**	**(N = 51)**	
Decreased frequency or amount	55.0 (22)	62.7 (32)	0.01
Increased frequency or amount	10.0 (4)	15.7 (8)	NS
Type of drugs preferred	35.0 (14)	13.7 (7)	NS
Not specified	-	7.8 (4)	NS

*NS not significant (p>0.05).

## Discussion

This study documents for the first time the characteristics of relapse following abstinence-oriented drug treatment among PWUD in Bangladesh. The data clearly show that relapse is common, occurring within 1 month of discharge from the treatment facility in most cases. The relapse rates documented in this study are comparable to those from developed countries, which show 60% or more relapse amongst heroin users [16,22]. However, the relapse rates recorded here occurred following treatment in clinics by a standardised, structured approach that combines detoxification, rehabilitation and training on different behavioural aspects in a 3-month programme. It is not known whether these results can be generalized to PWUDs who undergo treatment in other clinics and programmes in Bangladesh. Furthermore, although it is likely that the numbers of PWUDs who relapsed would have increased if they had been followed up for longer than 2 months after discharge, it is not clear whether the rate of relapse would have increased over time. Other studies have shown that the initial few post-treatment months are the most vulnerable period for relapse [16,22,23].

This study also demonstrates that relapse is more common among female PWUDs. Moreover, most women who were enrolled in this study had previously undergone treatment and therefore already had a history of relapse. We found that these women who had previously undergone treatment had almost twice the risk of relapsing again compared with those who had not. From this study it was not possible to gauge the reasons for repeated drug treatment failures. However, according to anecdotes of service providers, female PWUDs who sell sex seek treatment repeatedly because their ability to attract clients of commercial sex deteriorates with prolonged drug use: once treated, they are again attractive to clients. The finding that female PWUDs who sold sex after discharge were 2.5 times more likely to relapse than those who did not may support this possibility, but alternatively could reflect the need to earn money for drugs by selling sex. Injection of pharmaceuticals was less common among female than male PWUDs, most women preferring to smoke heroin; this has been shown in previous studies [1,4,7]. It is believed that relapse is generally more common among those who use heroin than among those who use other drugs [22], which could explain why the women enrolled in this study had higher relapse rates than the men.

For both male and female subjects, where they lived and who they lived with before admission to the clinic correlated with relapse rates: for men, the relevant factors were living with peer groups of PWUDs and alone and for women unstable living conditions. These structural factors indicate enhanced vulnerability of subjects who lack family support and are lonely, isolated or supported only by other PWUDs, factors that may serve to reinforce drug-taking behaviours. Analysis of post-discharge factors produced a similar finding in that men returning to an environment of living alone and in unstable housing were more likely to relapse. The importance of estrangement from families is further supported by the findings that for a large proportion of PWUDs, the primary reason for relapse was pressure from PWUD friends or sexual partners. Several studies have shown that poor, unstable living conditions promote risky behaviours with regard to drug use because they potentially expose PWUDs to high rates of violence, sexual abuse and other vulnerabilities [24-26].

In the OST pilot project implemented in Bangladesh in 2010, of the 180 PWIDs enrolled only eight were female; four of them dropped out. The reasons for dropping out were that their male injecting partners had dropped out and the distance between the OST clinic and where they lived (unpublished data). Although it is difficult to generalise from these small numbers, the contexts appear to be similar to those of female PWUDs who drop out of or relapse from drug treatment programmes. The small numbers of women enrolled in the OST pilot study are mainly because it accepts only injectors: most female PWUDs are primarily smokers.

The finding that men who were not having non-commercial sex before admission are at enhanced risk of relapse reinforces the importance of a supportive family and happy conjugal life. For women, the enhanced risk of relapse associated with not having children to support may also be a reflection of not having a family: both the emotional support and responsibilities associated with having a family can work as deterrents to living a chaotic life [25,27]. Although the NGO-run treatment programmes have provisions for family counselling, it is not always possible to provide this. Moreover, childcare facilities or pregnancy-related services are completely absent from these NGO-run programmes. Conversely, PWIDs attending the OST pilot for at least 1 year showed remarkable improvement in their family life and overall quality of life (unpublished data).

In addition to assessing differences in factors associated with relapse among male and female PWUDs, this study highlights other differences between men and women who access drug treatment. Many female PWUDs enrolled in this study sold sex, had multiple sex partners, often concomitantly, and used condoms less frequently with their commercial partners than did the male PWUDs. Such risk behaviours have been documented before from Bangladesh [7,9]. All published data from these studies confirm that female PWUDs require harm reduction services both in regard to drugs and to promoting safe sex. The vulnerability of male PWUDs was also high as shown by the high rates of imprisonment and the known correlation between imprisonment and HIV infection [28-30]. Moreover, to earn money, more male than female PWUDs sold blood. This is of particular concern because a large proportion of the men enrolled in our study were injectors and the national sero-surveillance data of 2007 showed that 54% of male PWIDs in Dhaka had antibodies to hepatitis C [11,31].

This study was conducted prior to the introduction of the OST pilot programme in Bangladesh. Experience and information gained from the OST pilot has led to the Ministries of Health and Home of the Government of Bangladesh to acknowledge that OST using methadone is effective not only in terms of harm reduction but as treatment. Repeated attempts at drug treatment and high relapse rates make both male PWIDs and female PWUDs ideal candidates for OST. The availability of OST for those who use opioids but do not necessarily inject would no doubt improve the situation given the evidence available from other countries such as the Netherlands [12]. Thus, it is important to develop linkages between the drug treatment and OST clinics in the future.

## Conclusions

Relapse after frequent (standardised) abstinence-oriented inpatient treatment in a Bangladesh sample, with opioid dependence and without OST, is extremely high. There are gender differences and social factors play a role in relapse. Drug dependence is recognised to be a multi-factorial health disorder that often follows the course of a relapsing and remitting chronic disease [32]. To enhance the coverage and maximise the outcomes of drug treatment strategies in Bangladesh, a comprehensive approach with psychosocially assisted pharmacological treatment should become available and accessible for PWUDs [33,34]. Especially for poor and marginalized PWUDs, this would address diverse issues, in a gender-sensitive manner.

## Competing interests

All authors declare there is no conflict of interests. Yuki Maehira was the doctorate-degree student of Kyoto University Graduate School of Medicine, Japan, when the Heiwa Nakajima Foundation (HNF) fund was provided for the data gathering portion of the study implemented in Dhaka, Bangladesh, while the HNF had no further role in the study design; in the collection, analysis and interpretation of data; in writing of the report; or in the decision to submit the paper for publication.

## Authors’ contributions

YM and TA designed the study, wrote the protocol and TA supervised all process of the study implementation. EIC managed to coordinate and supervise field data collection. MR supervised statistical analyses. RD, TKG, IM and SA supported timely data collection at clinics and outreach to study population in the field. NT provided advices of behavioural epidemiology for YM to draft the manuscript. All authors contributed to and have approved final manuscript.
